# 

**Published:** 1911-02-04

**Authors:** 


### CONTENTS.

| | PAGE |
|---|---|
| **Medical Fees** | 547 |
| **An Excellent Example** | 548 |
| **Annotations—** The Social Value of the Premature—The Doctor as Patient—A Medical Composer—Turkish Court Physicians | 549 |
| **Medical Opinion and Movement** | 550 |
| **Hospital Clinics—** Skin Affections of the Beard Region due to Micro-organisms, and their Treatment. By H. G. Adamson, M.D. | 553 |
| **Practical Notes on Diagnosis and Treatment** | 556 |
| **The Royal Army Medical Corps Section—** The Lack of Calcium Salts in the Food of Troops on Active Service as a Predisposing Cause of Enteric Fever and Dysentery... | 557 |
| **News and Events of the Week** | 559 |
| **Editor's Letter-Box—** Research Defence Society | 560 |
| **Obituary** | 560 |
| **The Week's Work—** A Record of Progress at Leeds—The Critic's Reward—Death Certification in Hospitals—An Atrocious Calumny—The Harm of Hospital Scares—Surprise Visits to Hospitals | 561 |
| **Special Institutional Articles—** Notes on Hospital Planning | 563 |
| The Lighting of Hospitals | 565 |
| **The Hospital World** | 566 |
| **Institutional Notes and News** | 567 |
| **Gifts and Bequests** | 569 |
| **Jottings** | 570 |
| **Institutional Letter-Box—** The Norfolk and Norwich Hospital | 571 |
| **New Appliances and Things Medical** | 571 |
| **Building Works Contemplated and in Progress** | 571 |
| **The Week's Novelties** | 571 |
| **"Bovril" Specialties** | 572 |

